# Long-Latency Somatosensory Evoked Potentials of the Subthalamic Nucleus in Patients with Parkinson’s Disease

**DOI:** 10.1371/journal.pone.0168151

**Published:** 2017-01-12

**Authors:** Carlos Trenado, Saskia Elben, Lena Friggemann, Sonja Gruhn, Stefan Jun Groiss, Jan Vesper, Alfons Schnitzler, Lars Wojtecki

**Affiliations:** 1 Institute of Clinical Neuroscience and Medical Psychology, Medical Faculty, Heinrich Heine University, Duesseldorf, Germany; 2 Department of Neurology, University Clinic, Heinrich Heine University, Duesseldorf, Germany; 3 Department of Functional and Stereotactic Neurosurgery, University Clinic, Heinrich Heine University, Duesseldorf, Germany; University of Toronto, CANADA

## Abstract

Somatosensory evoked potentials (SSEPs) are a viable way to measure processing of somatosensory information. SSEPs have been described at the scalp and the cortical level by electroencephalographic, magnetoencephalographic and intracranial cortical recordings focusing on short-latency (SL; latency<40 ms) and long-latency (LL; latency>40 ms) SSEPs as well as by deep brain stimulation (DBS) electrode studies targeting SL-SSEPs. Unfortunately, LL-SSEPs have not been addressed at the subcortical level aside from the fact that studies targeting the characteristics and generators of SSEPs have been neglected for the last ten years. To cope with these issues, we investigated LL-SSEPs of the subthalamic nucleus (STN) in twelve patients with Parkinson’s disease (PD) that underwent deep brain stimulation (DBS) treatment. In a postoperative setting, LL-SSEPs were elicited by median nerve stimulation (MNS) to the patient’s wrists. Ipsilateral or contralateral MNS was applied with a 3 s inter-stimulus interval. Here, we report about four distinctive LL-SSEPs (“LL–complex” consisting of P80, N100, P140 and N200 component), which were recorded by using monopolar/bipolar reference and ipsi/contralateral MNS. Phase reversal and/or maximum amplitude provided support for the generation of such LL-SSEPs within the STN, which also underscores a role of this subcortical structure in sensory processing.

## Introduction

Deep brain stimulation (DBS) is an established treatment for Parkinson’s disease (PD) [1, 2], albeit the exact mechanism on how DBS operates to improve PD symptoms remains unclear. Nevertheless, different lines of research suggest that DBS modulates the oscillatory activity of pathological networks [3]. At present, the preferred target for DBS in PD is the subthalamic nucleus (STN) which has been implicated in movement, cognition and sensory processing [4, 5, 6]. Focusing on sensory processing, somatosensory evoked potentials (SSEPs) elicited by median nerve stimulation (MNS) have been proposed as an objective examination method for studying nerve function with potential to provide information about the spinal cord, the cortex and subcortical regions such as the STN [7].

According to their latency, SSEPs can be classified as short-latency (SL) and long-latency (LL) potentials [7, 8, 9]. On the one hand, SL-SSEPs possess latencies ranging from 11 ms to approximately 40 ms. SL-SSEPs have been studied at the scalp, the cortical and the subcortical level and shown to be stable under anesthesia effects [7]. Moreover, these potentials are commonly used in clinical procedures including diagnosis of lesions in the white matter of the brain (especially the primary somatosensory cortex), the spinal cord and for intraoperative monitoring [10]. On the other hand, LL-SSEPs possess latencies greater or equal to 40 ms. LL-SSEPs have been addressed at the scalp and the cortical level but not at the subcortical level. To cope with this, we addressed LL-SSEPs in the STN of PD patients. We aim to provide a characterization of such potentials in terms of amplitude, phase reversal and reproducibility. In turn, such aspects could give insight into the involvement of the STN in somatosensory processing within the context of PD.

Previous studies focusing on LL-SSEPs at the scalp and the cortical level (intracranial) reported about complexes such as P45-N60 over the contralateral somatosensory cortex, N30-P65-N70 over the frontal cortex, a bilateral frontal complex P80-P100 followed by the complex N140-N260-P200-P300-P420-N360-N460, including LL-SSEP components such as P45, N60, P100 with the cortical peri-central P50 and the pre- and post-central derived N80-P80 with corresponding generator as the contralateral area 3b of the somatosensory cortex [8, 9, 10, 11, 12]. In addition, negative components around 65 ms or 77.9 ms as well as positive components around 94 ms or 111.2 ms have been recorded by using intracortical electrodes stereotactically implanted in the anterior subdivision of supplementary motor areas (the pre-SMA) [13, 14], while a negative-positive biphasic complex (N60-P90) was recorded in the upper bank of the Sylvian fissure (SII area) [15].

At the subcortical level, previous DBS electrode studies focused on SL-SSEPs in the STN [16], the zona incerta (ZI) [17] and thalamic/subthalamic regions [18, 19, 20, 21]. Specifically, the N18 and P18 in the STN, the P16 in the subthalamic region and the zona incerta (ZI), the biphasic component P13-N16 with highest amplitude in the medial lemniscus, thalamic and STN SSEPs with latencies (17.9±1.7 ms) and (18.2 ±1.5 ms), a monophasic negative component with latency (17.3±2.2 ms) and maximum amplitude in the ventral intermediate thalamus (VIM), a high-amplitude positive component at (15.5 ms) in the ventrocaudal thalamus (Vc) and a low-amplitude triphasic (P-N-P) complex with first positive (13.3 ms) and negative (16 ms) components located in the VIM.

In the present study we hypothesized that LL-SSEPs are generated in the STN by assuming it is a global integrative structure [22] with neurons responsive to somatosensory stimuli mainly present in its dorsolateral region [23]. From a clinical perspective, it has also been documented that placement of DBS electrodes within the STN in locations with identified kinesthetic cells is correlated to good clinical outcomes and that patients experience transient paresthesia during STN-DBS stimulation onset which is different from stimulation effects of the internal capsule [24, 25]. Thus, the STN seems to be involved in sensory processing and represents a good candidate in the generation of LL-SSEPs.

## Materials and Methods

### Patients

Twelve PD patients (4 female and 8 male, mean height: 1.72±0.078 m) participated in this study. Only patients with normal surface EEG median nerve somatosensory evoked potentials (N20 latencies and amplitudes in reference to our clinical neurophysiological values derived from a control group by scalp electroencephalography, specifically N20 maximum latency of 22.1 ms for a group whose mean height is 1.72 m and minimum amplitude greater or equal to 1.5 μV) during pre-surgical clinical evaluation were enrolled in the study. Furthermore, patients with cognitive impairment and other diseases that may have impaired their somatosensory processing such as stroke and leukoencephalopathy were excluded. The mean age of patients was 61.2±4.9 years; range: (55,72) and the mean disease duration at the time of the surgery was 12.2±4.6 years; range: (5,21). The entire group fulfilled the clinical criteria for the indication of deep brain stimulation (DBS). Table 1 gives an overview of age, gender, duration of illness at the time of surgery, hemisphere preference and the score of the motor part of the UPDRS *on* and *off* L-Dopa. The study was in compliance with the Helsinki Declaration and was approved by the ethics committee of the University Hospital Düsseldorf. Patients that participated in the study signed an informed consent form.

**Table 1:** Patient Collective: m = male, f = female, Age (at the time of operation), Duration since diagnosis (number of years since disease diagnosis). Score of the motor part of the Unified Parkinson’s Disease Rating Scale (UPDRS) without medication: UPDRS *Off*, score of the motor part of the UPDRS with medication: UPDRS *On*. The UPDRS test was performed prior to surgery.

Patient	Gender	Age (years)	Duration since diagnosis (years)	Dominant Side	UPDRS *Off/On*
1	m	55	8	right	33/16
2	m	64	11	right	35/19
3	f	64	21	left	32/12
4	m	62	16	left	36/11
5	f	62	10	left	35/13
6	m	57	8	left	31/15
7	f	55	15	left	45/21
8	m	60	15	right	35/4
9	m	55	15	right	33/13
10	m	72	14	right	33/8
11	m	62	8	left	49/26
12	f	60	5	right	14/7

### Electrode implantation

Oral antiparkinsonian medications were stopped the day before surgery and if applicable patients were administered a temporary apomorphine pump (S1 Table). All patients underwent a bilateral implantation of DBS electrodes in the STN. Implantation took place under local anesthesia. Surgery was performed in either the Department for Stereotactic and Functional Neurosurgery of the University Hospital Duesseldorf or the Clinic for Stereotactic and Functional Neurosurgery of the University Hospital Cologne. The stereotactic frame used during the operation was the modified Riechert-Mundinger System, MRC Systems, Heidelberg. The STN was localized by using MRI scans fused with stereotactic CT prior to surgery. The final DBS electrode location within the STN was determined by Multiunit Microelectrode Recordings (MER) activity patterns and the best clinical effect during test stimulation. The system used for MER was the ISIS MER System (Inomed, Medizintechnik GmbH, Emmendingen, Germany), which consists of a lead cable (ISIS MER lead cable), a preamplifier (ISIS MER preamp), the ISIS MER headbox, EMG electrode lead cables and a computer unit for data acquisition equipped with the ISIS MER software (version 2.4). The DBS electrodes used were Medtronic 3389 (DBS lead model 3389, Medtronic GmbH, Minneapolis, USA). Each electrode had four contacts (zero, one, two and three) possessing a length of 1.5 mm and a circumference of 1.27 mm. The distance between contacts was 0.5 mm.

### Recording and stimulation

All patients were tested one day after surgery. For MNS, the OSIRIS Neuro Stimulator (Inomed, Medizintechnik GmbH, Emmendingen, Germany) was utilized together with electrodes placed over the median nerve at the wrist. MNS was applied bilaterally (alternating between both sides: ipsi- and contralateral) and consisted of constant-current square pulses of 200 μs presented with an interstimulus interval of 3 s. The current intensity was adjusted for each patient just above the motor threshold. A total of 240 stimuli were delivered (120 for each side) over approximately 12 minutes.

Postoperative recordings were performed through externalized extensions of the final DBS electrodes, which were connected to an EEG head-box (Brain Products, Munich, Germany) that was connected to a recording computer equipped with the BrainVision Recorder software (Version 1.03, BrainAmp MR-Plus, Brain Products, Munich, Germany). Reference and ground electrodes were respectively attached to the forehead (frontomedian reference) and the clavicle (right side) of each patient. The recording parameters were set-up as follows: *Low Cutoff* filter of 0.5 Hz, *High Cutoff* filter of 2000 Hz and a *sampling rate* of 10 kHz. The recording was performed in a separate, quiet room and patients were tested in medication “ON” (L-dopa) without agonists (see list of medications and doses for each patient prior to surgery in S2 Table). Patients were sitting comfortably and had their eyes closed.

### Retrospective control of recoding localization

In order to visualize the coordinates of individual contacts of the final electrode and the recording location, macroelectrodes were visualized in the Schaltenbrand atlas [26] by using the electronic clinical brain atlas. The coordinates of the final electrode were determined by fusion of postoperative CT with preoperative stereotactic MRI. The coordinates of the respective contact showing phase reversal and/or maximum amplitude were calculated and normalized for visualization on the Schaltenbrand atlas. The obtained coordinates were averaged across individual LL-SSEP components and by considering stimulation side (ipsi- and contralateral). Please read in the discussion some limitations on estimating such coordinates, which are available upon request by contacting the corresponding author.

### Data analysis

All data were filtered and analyzed with the BrainVision Analyzer 2 (Version 2.0, Brain Products, Münich Germany). Data were visually screened and artifacts rejected. Individual electrode contacts were referenced with respect to a frontal electrode placed on the forehead (monopolar data) or with respect to other contacts: zero vs. one, one vs. two, two vs. three (bipolar data). For monopolar and bipolar data, the contact with the highest amplitude (peak-to-peak) was selected for further analysis: First, a 50 Hz *Notch* filter was applied; Second, data was divided into segments (starting at 100 ms before stimulus onset and ending at 1000 ms after stimulus onset) which were baseline corrected between -100 and 0 ms; Third, segments were filtered with a *High Cutoff* filter 40 Hz and subsequently averaged according to electrode’s reference (monopolar and bipolar) and stimulation side (ipsi- and contralateral). Note that such filtering procedure could lead to effects on early SSEP components (e.g. the N30) and eliminate frequency components that potentially contribute to cortical SSEP generators [27].

### Statistical analysis

We made use of t-test for unpaired samples when comparing latencies of SL-SSEPs in the present study and results reported in the literature. For the cases in which the assumption of normality was violated (Kolmogorov-Smirnov) non-parametric tests were utilized. Specifically, we used the Wilcoxon signed-rank test with a significant level of 5% to compare latencies between ipsi- and contralateral stimulation of monopolar and bipolar data. The same test was applied to compare the latencies of LL-SSEPs of each stimulation side between monopolar and bipolar data. Statistical analysis was performed by using the software IBM SPSS Statistics (Version 23, IBM Software, Business and analytics, Armonk, NY, USA).

## Results

**Fig 1:**
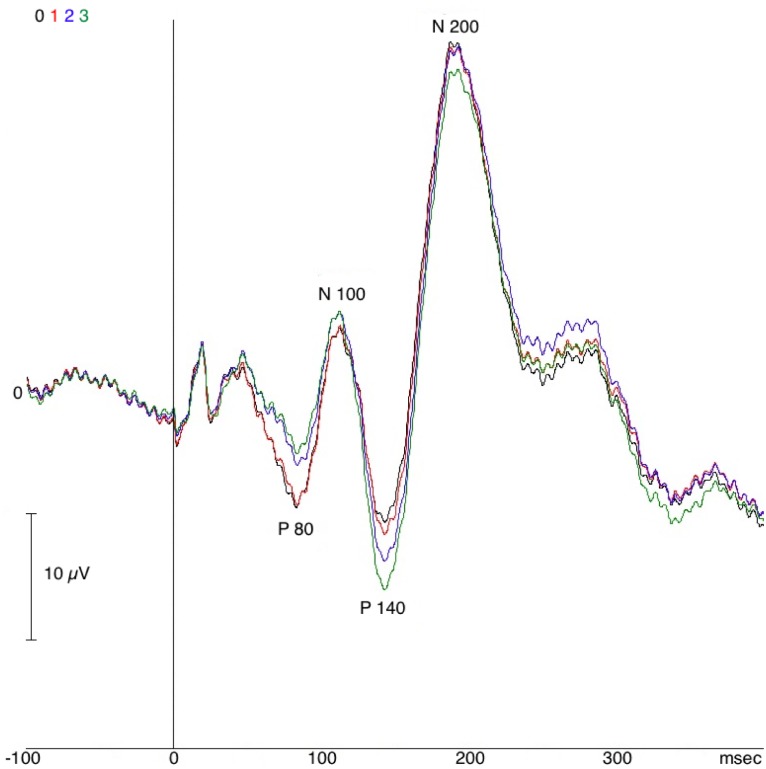
Example of long-latency somatosensory evoked potential (LL-SSEP) Complex, Patient 4, right STN, monopolar reference, contralateral median nerve stimulation (MNS). Black: Contact 0, red: Contact 1, blue: Contact 2, green: Contact 3.

For each STN (twenty four), LL-SSEPs were elicited by ipsilateral and contralateral MNS and were visible by monopolar and bipolar contacts. Specifically, four distinctive SSEP components were found delineating a “LL-complex”: P80, N100, P140 and N200 (Fig 1).

### Monopolar reference

**Fig 2:**
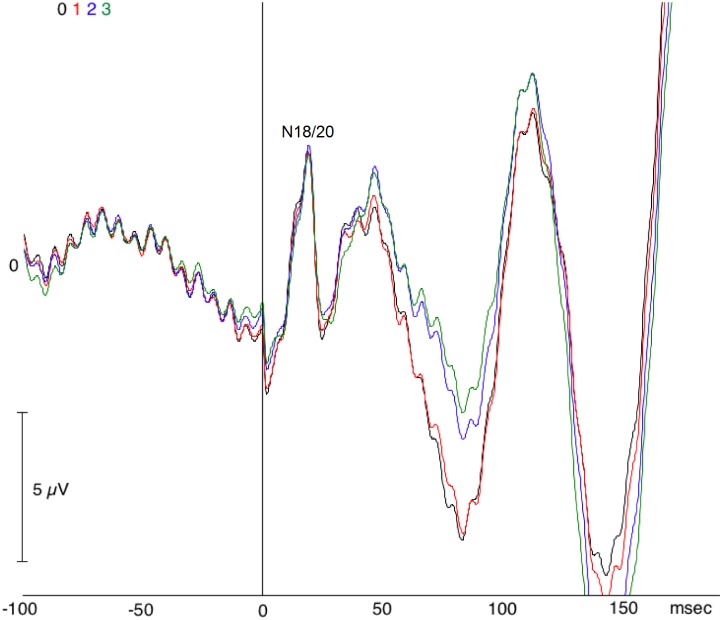
Zoom-in view of Fig 1 emphasizing the short-latency somatosensory evoked potential (SL-SSEP) component N20: Patient 4, right STN, monopolar reference, contralateral median nerve stimulation (MNS). Black: Contact 0, red: Contact 1, blue: Contact 2, green: Contact 3.

With regard to SL-SSEPs, a component with mean latency 18.9±1.2 ms and mean amplitude 2.7±1.9 μV was elicited in twenty-two out of twenty four STN’s. We name this component as N18/20 by considering that its latency corresponds to N18 as documented by previous studies [16, 18] and based on its correlation with the scalp N20 as previously reported [16] (See (Fig 2) for an example of this component). Statistical comparison of mean latencies (t-test for unpaired samples) between the N18/20 of the present study and data reported in the literature revealed no significant difference (Table 2). In reference to our clinical neurophysiological values derived from a control group by surface EEG recordings, the patients in the present study showed N18/20 latencies (range: (17.7, 20.1)) below the N20 latency (22.1 ms) for a control group with mean height 1.72 m, while most of the N18/20 amplitudes (range: (0.8, 4.6)) were above the N20 amplitude of 1.5 μV in agreement with our specified inclusion criteria.

**Table 2:** Statistical comparison of mean latencies between the N18/20 in the present study and data reported in the literature revealed no significant difference (significance level of 0.05). N denotes the number of nuclei with occurrence of the N18/20, Latency (ms): Mean ± standard deviation (SD) (range), Amplitude (μV): Mean ± SD (range), NA “not available”. This finding provides support for the used methodology, namely forehead rather than linked earlobes/mastoids for monopolar reference and filtering methods (Notch and 40 Hz high cut-off), in eliciting somatosensory evoked potentials (SSEPs).

	Present study	Pesenti et al. 2003	Hanajima et al. 2004	Klostermann et al. 2003
**Latency(ms)**	18.9±1.2(17,22)	18.0±2.3(13.7,20.9)	18.2±1.5(NA)	18.7±1.4(NA)
**Amplitude(μV)**	2.7±1.9(0.4,8.2)	0.7±0.3(0.3,1.2)	NA(NA)	1.1±0.4(NA)
**N**	22	11	22	11
**p-value**		0.1468	0.0948	0.6722

The recorded LL-SSEPs did not show phase reversal. LL-SSEPs were reproducible and already visible after one to five MNS repetitions. There was no significant difference in latency between ipsi- and contralateral stimulation in none of the four LL-SSEP components according to the Wilcoxon signed-rank test (p = 1 for P80, p = 0.894 for N100, p = 0.666 for P140, p = 0.932 for N200). The N200 was the most consistent LL-SSEP wave, as it was elicited in each of the considered STNs and had the highest mean amplitude. The second most common LL-SSEP wave was the P140, which showed the second highest mean amplitude (Table 3).

**Table 3:** Long-latency somatosensory evoked potential (LL-SSEP) components corresponding to monopolar data: N denotes the number of nuclei with a referred wave, Latency (ms): Mean ± SD (range), Amplitude (μV): Mean ± SD (range).

Wave	Stimulation(Side)	N	Latency(ms)	Amplitude(μV)
P80	Contralateral	19	80.9±11.3(61,107)	9.5±5.8(1.6,19.7)
P80	Ipsilateral	16	78.5±10.4(60,93)	6.7±5.2(1.4,16.7)
N100	Contralateral	20	104.8±13.8(76,122)	8.3±4.8(2,21.5)
N100	Ipsilateral	19	100.8±11.9(77,120)	7.2±4.7(1.8,20.4)
P140	Contralateral	23	139.4±16.8(121,176)	12.2±7.4(1.8,28)
P140	Ipsilateral	23	140.0±16.0(118,175)	14.0±7.0(0.7,29.9)
N200	Contralateral	24	200.4±24.0(172,266)	17.6±11.3(1.1,41.9)
N200	Ipsilateral	24	198.3±20.6(170,258)	18.6±11.2(1.5,49.7)

### Bipolar reference

There was no significant difference in latency between ipsi- and contralateral stimulation in none of the four LL-SSEP components according to the Wilcoxon signed-rank test (p = 0.889 for P80, p = 0.798 for N100, p = 0.139 for P140, p = 0.875 for N200).

**Fig 3:**
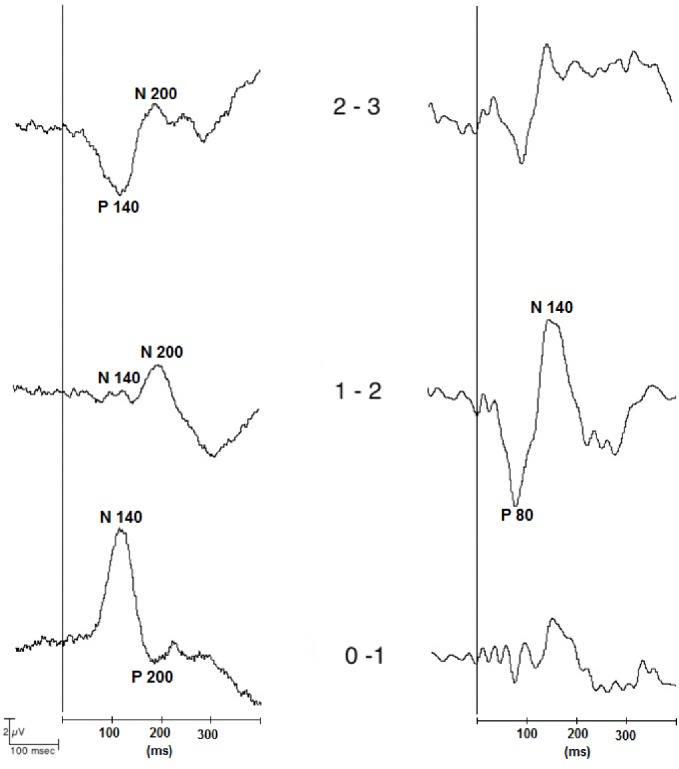
Example of LL-SSEPs recorded with bipolar reference. Left: Phase reversal of the P140 and the N200, Patient 2, left STN, ipsilateral MNS. Right: Maximum amplitude of the P80 and N140, contact (1 vs. 2), Patient 4, right STN, contralateral MNS.

Phase reversal of LL-SSEP components was observed although not simultaneously in all bipolar contacts (Table 4), as exemplified by the P140 and the N200 in (Fig 3, left).

**Table 4:** LL-SSEP components corresponding to bipolar data: N denotes the number of nuclei with a referred wave, Latency (ms): Mean ± SD (range), Amplitude (μV): Mean ± SD (range), “Total Phase reversals” denotes the total number of reversals across bipolar contacts for each LL-SSEP component and stimulation side, “Max 1–2” denotes the number of nuclei with maximum amplitude at bipolar contact (1 vs. 2).

Wave	Stimulation(Side)	N	Latency (ms)	Amplitude (μV)	Total Phase reversals	Max 1–2
P80	Contralateral	13	83.4± 10.6)(65,109)	4.7±4.4(1.1,15.7)	7	5
P80	Ipsilateral	11	81.7±6.7(70,92)	4.6±7.0(0.7,23.7)	3	4
N100	Contralateral	12	100.0±14.6(79,124)	3.6±2.4(0.6,8.2)	9	2
N100	Ipsilateral	13	98.7±14.4(78,120)	2.3±1.3(0.8,4.4)	5	5
P140	Contralateral	13	133.6±8.7(122,150)	3.7±2.4(1.1,8.3)	7	2
P140	Ipsilateral	20	137.1±17.6(114,178)	4.7±7.1(1.1,33.6)	5	6
N200	Contralateral	15	195.3±15.1(176,228)	7.1±4.9(1.7,17.6)	6	3
N200	Ipsilateral	21	195.8±20.8(162,251)	4.4±4.0(0.9,16.4)	7	4

**Fig 4:**
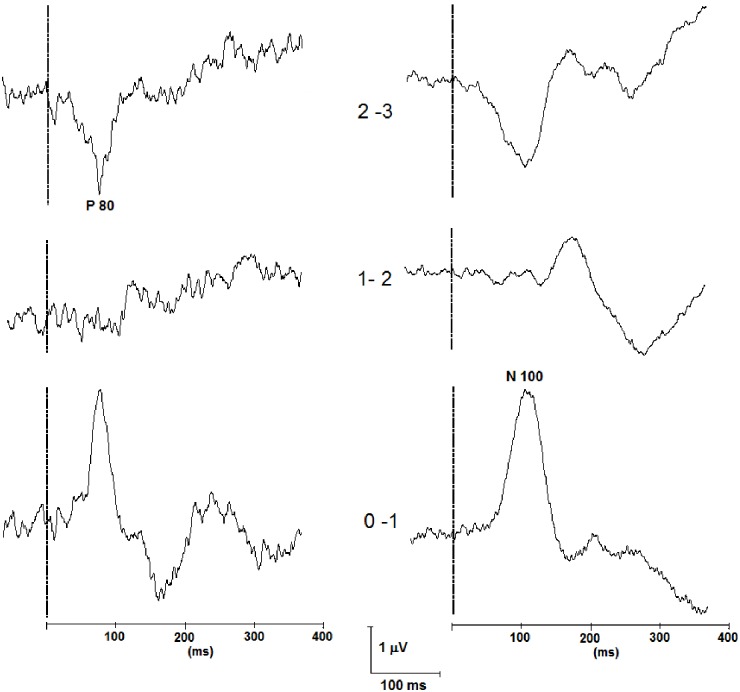
Example of LL-SSEPs recorded with bipolar reference. Left: Phase reversal of the P80 at contact (0 vs. 1), Patient 9, right STN, ipsilateral MNS. Right: Phase reversal of the N100 at contact (2 vs. 3), Patient 2, left STN, contralateral MNS.

Examples of phase reversal of the P80 and the N100 can be seen in Fig 4. The number of phase reversals for each bipolar contact is provided in Table 5.

**Table 5:** Number of phase reversals for each bipolar contact, LL-SSEP component and stimulation side (ipsi- and contralateral). No significant difference was found in the number of phase reversals between bipolar contacts according to the Wilcoxon signed-rank test (p = 0.157 for ((0 vs. 1) vs. (1 vs. 2)), p = 0.395 for ((1 vs. 2) vs. (2 vs. 3)), p = 1.0 for ((0 vs. 1) vs. (2 vs. 3))).

Wave	Stimulation(Side)	Phase reversal(0 vs. 1)	Phase reversal(1 vs. 2)	Phase reversal(2 vs. 3)
P80	Contralateral	2	2	3
P80	Ipsilateral	1	1	1
N100	Contralateral	3	3	3
N100	Ipsilateral	1	1	3
P140	Contralateral	2	4	1
P140	Ipsilateral	2	2	1
N200	Contralateral	1	3	2
N200	Ipsilateral	3	3	1

Maximum amplitude of LL-SSEP components was observed in the bipolar contact (1 vs. 2), as exemplified by the P80 in (Fig 3, right). Both of the mentioned features occurred for contralateral and ipsilateral stimulation (Table 4). In the case of the N100 and the P140, the percentage of recordings with maximum amplitude corresponding to bipolar contact (1 vs. 2) was higher in the case of ipsilateral stimulation (ipsilateral vs. contralateral: (P80) 36.36% vs. 38.46%; (N100) 38.46% vs. 16.66%; (P140) 30%: vs. 15.38%; (N200) 19.04% vs. 20%).

### Monopolar vs. Bipolar reference

There was no significant difference in the latencies of LL-SSEPs between monopolar and bipolar data (Wilcoxon signed-rank test, significance level 5%).

## Discussion

This study focused on LL-SSEPs recorded postoperatively from the STN of PD patients through DBS electrodes. In contrast to previous studies addressing subcortical SL-SSEPs [18, 19, 20, 21], this is the first study reporting about LL-SSEPs in the STN. The four components described (e.g. P80, N100, P140 and N200) were elicited by both ipsi- and contralateral MNS. Phase reversal and/or maximum amplitude of LL-SSEP components were observed in bipolar contacts although not simultaneously in all of them, as exemplified by Fig 3 (contact (one vs. two)). The occurrence of SSEP’s phase reversal and/or maximum peak amplitude for contacts within the STN suggests that such potentials were locally generated in this structure. In support of this, retrospective localization of DBS electrodes by fusion of postoperative CT with preoperative stereotactic MRI as well as intraoperative MER activity patterns supported that coordinates of DBS electrodes were located within the STN. However, it is important to point out that the exact localization of such phase reversal using an atlas is limited due that: 1) estimation of the coordinates of such location should be better estimated along the axis of the macroelectrodes, 2) only three bipolar contacts are available for each electrode leading to poor spatial resolution, 3) accuracy errors are present when translating such estimations into the atlas coordinates.

Also, it is worth emphasizing that patients were tested under medication “ON” (L-dopa) without agonists to prevent their confounding effect in SSEPs. The elicited SL-SSEPs by using our recording approach were similar to the ones reported in previous studies [16, 18, 19].

Focusing on the generators of SL-SSEPs, previous studies directed towards the thalamus [18] and the VIM [20, 21] came to different conclusions regarding the generators of SL-SSEPs. This observation emphasizes the importance of additional studies to help clarify whether SSEPs have a generator within the basal ganglia network. Potential candidates for this purpose include areas within the STN, VIM or Vc.

Note that the occurrence of phase reversal and/or maximum amplitude of LL-SSEP components in different bipolar contacts precludes that all such components have the same generator within the STN or its connections, e.g. white matter pathways from cortical areas including somatosensory cortex. It is also important to note that although phase reversal of SSEPs is commonly regarded as an established approach for intraoperative localization of the sensorimotor cortex and the central sulcus [28, 29], our data and others [18, 30] favor the idea that such reversal feature is not only present at subcortical regions but may also represent a distinctive pattern characterizing neuronal activity at specific subcortical regions.

The four distinctive LL-SSEP components that we recorded were reproducible. They were already visible after only one MNS repetition and could be reproduced after only a few MNS repetitions. Since no significant difference between the mean latencies of monopolar and bipolar data was found, it can be assumed that the same potentials can be recorded by using monopolar and bipolar reference. Nevertheless, our finding about the non-significant difference cannot be safely interpreted based on the limited amount of data collected.

Unlike previous studies targeting LL-SSEPs at the scalp (the somatosensory cortex) and the cortical level (motor cortex) [8, 9, 10, 12, 31, 32, 33], we focused on subcortical structures, namely the STN of PD patients.

In particular some LL-SSEP complexes that have been addressed at the scalp and the cortical level include: P45-N60 over the contralateral somatosensory cortex, N30-P65-N70 over the frontal cortex, a bilateral frontal component P100 that is preceded by P80 and followed by a bilateral complex in the range between 100 and 500 ms e.g. N140-N260-P200-P300-P420-N360-N460, where N140-P200 is maximum over the vertex and the remaining components over the posterior regions [10]. Relevant LL-SSEP components in the range 40–250 ms include, P45, N60 and P100 with the cortical pericentral P50 and the pre- and post-central derived N80-P80 with corresponding generator as the contralateral area 3b of the somatosensory cortex [32]. These authors proposed that the scalp N120-P100 corresponds to the cortical N100-P100 while being generated in the supplementary motor cortex, and that N140 and P190 have bilaterally preferred generators which appear in the frontal lobes including the orbitofrontal cortex and the lateral and mesial frontal cortex. Nevertheless, one should be cautious by considering that a comparison between scalp and cortical potentials under different recording conditions (narcotics, age distribution of patients, different inter-stimulus intervals between groups) is problematic [11].

Our data, documenting the occurrence of LL-SSEPs in the STN, suggests that this subcortical structure takes part in somatosensory processing. Since LL-SSEPs were elicited by both ipsilateral and contralateral MNS, one could argue about feedback projections from the secondary somatosensory cortex (S2) to the STN via neighboring cortical areas, namely primary motor cortex (M1), the posterior parietal cortex (PPC) or precentral gyrus (PCG). This hypothesis is supported by the observation that subcortical and cortical LL-SSEP components present similar morphology and latencies when contrasting results from previous studies and our data. In addition, DBS-STN animal studies have demonstrated enhancement in neuronal activation in motor and sensory cortices presumably as a reinforcement mechanism involving the overlap between feedback and feed-forward responses along the basal ganglia-thalamo-cortical loop [34].

One should also note that a simultaneous cortical-subcortical recording is required for a strict comparison between cortical and subcortical SSEPs. Such recording could also give us deeper insight into a prospective cortico-subcortical somatosensory network.

In conclusion, the occurrence of LL-SSEPs in the STN of PD patients underscores a role of this structure in sensory processing. Due to the strategic involvement of the STN in the basal ganglia- thalamo-cortical loop, STN is suggested to play a sensorimotor integrative role.

## Supporting Information

S1 Table Flow rate of apomorphine pump for each patient. In particular, the syringes of the pump were filled with 2 APO-go^®^ ampoules so that 10 ml apomorphine (10 mg/ml)) were administered in total. It is worth emphasizing that L-Dopa test was taken at an inpatient stay before the operation. Thus the values of L-dopa-tests are considered to be independent of apomorphine in the pump.
(DOCX)

S2 Table Regular medications and doses for each patient prior to DBS surgery. Note that medications for each patient were restricted to L-Dopa without agonists the day of recording after its withdrawal for DBS electrode implantation surgery.
(DOCX)
