# Correction: Long-Latency Somatosensory Evoked Potentials of the Subthalamic Nucleus in Patients with Parkinson's Disease

**DOI:** 10.1371/journal.pone.0175602

**Published:** 2017-04-06

**Authors:** Carlos Trenado, Saskia Elben, Lena Friggemann, Sonja Gruhn, Stefan Jun Groiss, Jan Vesper, Alfons Schnitzler, Lars Wojtecki

There are multiple errors throughout the paper. In the second paragraph of the introduction, reference [10] is incorrectly placed after the third sentence. It should be placed after the fourth sentence and read as follows: On the other hand, LL-SSEPs possess latencies greater or equal to 40 ms [10].

The first sentence of the Monopolar reference section is missing the phrase “by controlateral MNS,” after the word elicited. The correct sentence is: With regard to SL-SSEPs, a component with mean latency 18.9±1.2 ms and mean amplitude 2.7±1.9 μV was elicited by contralateral MNS in twenty-two out of twenty four STN’s.

The first sentence of the third paragraph within the Discussion section incorrectly references the VIM as being independent from the thalamus. The correct sentence is: Focusing on the generators of SL-SSEPs, previous studies directed towards the thalamus [18] and its VIM [20, 21] came to different conclusions regarding the generators of SL-SSEPs.

## References

[1] Trenado C, Elben S, Friggemann L, Gruhn S, Groiss SJ, Vesper J, et al (2017) Long-Latency Somatosensory Evoked Potentials of the Subthalamic Nucleus in Patients with Parkinson’s Disease. PLoS ONE 12(1): e0168151 doi: 10.1371/journal.pone.0168151 28081139 10.1371/journal.pone.0168151 PMC5231369

