# Supplementary material for: Long-Latency Somatosensory Evoked Potentials of the Subthalamic Nucleus in Patients with Parkinson’s Disease
Source: PLoS One. 2017 Jan 12;12(1):e0168151. doi: 10.1371/journal.pone.0168151 (PMC5231369; doi:10.1371/journal.pone.0168151)
Supplement: S1 Table — In particular, the syringes of the pump were filled with 2 APO-go® ampoules so that 10 ml apomorphine (10 mg/ml)) were administered in total. It is worth emphasizing that L-Dopa test was taken at an inpatient stay before the operation. Thus the values of L-dopa-tests are considered to be independent of apomorphine in the pump. (DOCX) [file pone.0168151.s001.docx]

| **Patient** | **apomorphine pump (flow rate)** |
| --- | --- |
| 1 | 0.5 ml/h (2.5 mg/h) |
| 2 | 0.5 ml/h (2.5 mg/h) |
| 3 | 0.7ml/h (3.5 mg/h) |
| 4 | 0.3 ml/h (1.5 mg/h) |
| 5 | 0.3 ml/h (1.5 mg/h) |
| 6 | Not used |
| 7 | Not used |
| 8 | 0.6 ml/h (3.0 mg/h) |
| 9 | 1.0 ml/h (5.0 mg/h) |
| 10 | Not used |
| 11 | 0.3 ml/h (1.5 mg/h) |
| 12 | 0.6 ml/h (3.0 mg/h) |
