# Supplementary material for: Long-Latency Somatosensory Evoked Potentials of the Subthalamic Nucleus in Patients with Parkinson’s Disease
Source: PLoS One. 2017 Jan 12;12(1):e0168151. doi: 10.1371/journal.pone.0168151 (PMC5231369; doi:10.1371/journal.pone.0168151)
Supplement: S2 Table — Note that medications for each patient were restricted to L-Dopa without agonists the day of recording after its withdrawal for DBS electrode implantation surgery. (DOCX) [file pone.0168151.s002.docx]

| **Patient** | **Regular medications** |
| --- | --- |
| 1 | Amantadine 100 mg,Carbidopa/Levodopa/Entacapone 150 mg, Pramipexole 0.35 mg, Levodopa/Carbidopa 200 mg, Domperidone 2 ml |
| 2 | Levodopa/Benzerazid LT, Levodopa/Carbidopa 100 mg, Levodopa/Carbidopa retard 200 mg, Pramipexole 0.35 mg, Rasagiline 1 mg, Mirtazapine 15 mg, Verapamil 80 mg, Domperidone |
| 3 | Levodopa/Benzerazid LT, Levodopa/Benzerazid 125 mg, Carbidopa/Levodopa/Entacapone 100 mg, Rotigotine 4 mg, Levodopa/Carbidopa retard 200 mg, Domperidone 10 mg |
| 4 | Levodopa/Benzerazid 125 mg, Levodopa/Benzerazid LT, Levodopa/Benzerazid Depot, Ropinirole Hcl 1 mg, Amantadine 100 mg, Domperidone 20 mg, Esomeprazol 20 mg |
| 5 | Levodopa/Benzerazid 62.5 mg, Tolcapone 100 mg, Rasagiline 1 mg, Mirtazapine 30 mg, Tetrazepam, Domperidone 20 mg |
| 6 | Levodopa/Benzerazid 62.5 mg, Domperidone 20 mg, Rasagiline 1 mg, Pramipexole 0.7 mg |
| 7 | Levodopa/Benzerazid LT, Levodopa/Benzerazid Depot., Carbidopa/Levodopa/Entacapone 100 mg, Cabergoline 1mg, Aminophylline (Chronic bronchitis), Tetrazepam 50 mg, Tramadolhydrochlorid 100 mg, Mono-Mg-L-diglutamat, Levothyroxine 25 mg, Domperidone |
| 8 | Levodopa/Carbidopa 100 mg, Levodopa/Carbidopa retard 100 mg, Pramipexole 0.35 mg, Carbidopa/Levodopa/Entacapone 100 mg, Carbidopa/Levodopa/Entacapone 50 mg, Domperidone |
| 9 | Levodopa/Benzerazid 125 mg, Levodopa/Benzerazid LT, Levodopa/Benzerazid Depot., Pramipexole 0.35 mg, Entacapone 200 mg, Domperidone |
| 10 | Levodopa/Benzerazid LT, Levodopa/Benzerazid 125 mg, Levodopa/Benzerazid Depot., Tolcapone 100 mg |
| 11 | Cabergoline 3 mg, Levodopa/Benzerazid 62.5 mg, Ropinirole Hcl 5 mg |
| 12 | Levodopa/Carbidopa 100/25 mg, Levodopa/Carbidopa 150/37.5 mg, Levodopa/Benserazid LT 100/25 mg, Rasagiline 1 mg, Pramipexole 0.7 mg, Domperidone, Latanoprost ophtalmic, Diclofenac |
